# Is Imaging Bacteria with PET a Realistic Option or an Illusion?

**DOI:** 10.3390/diagnostics13071231

**Published:** 2023-03-24

**Authors:** Shashi B. Singh, Sadikshya Bhandari, Shisir Siwakoti, Rabi Bhatta, William Y. Raynor, Thomas J. Werner, Abass Alavi, Soren Hess, Mona-Elisabeth Revheim

**Affiliations:** 1 Department of Radiology, Hospital of the University of Pennsylvania, 3400 Spruce Street, Philadelphia, PA 19104, USA; 2 Kathmandu University School of Medical Sciences, Dhulikhel Hospital, Dhulikhel 45200, Nepal; 3 Universal College of Medical Sciences, Bhairahawa 32900, Nepal; 4 Department of Radiology, Rutgers Robert Wood Johnson Medical School, 1 Robert Wood Johnson Place, MEB #404, New Brunswick, NJ 08901, USA; 5 Department of Radiology and Nuclear Medicine, Hospital Southwest Jutland, 6700 Esbjerg, Denmark; 6 Department of Regional Health Research, Faculty of Health Sciences, University of Southern Denmark, 5230 Odense, Denmark; 7 The Intervention Center, Division of Technology and Innovation, Oslo University Hospital, 0424 Oslo, Norway; 8 Division for Radiology and Nuclear Medicine, Oslo University Hospital, 0424 Oslo, Norway; 9 Norway and Institute of Clinical Medicine, Faculty of Medicine, University of Oslo, 0315 Oslo, Norway

**Keywords:** PET, imaging, bacteria, infection, inflammation, [^18^F]FDG, radiotracers

## Abstract

The application of [^18^F]-fluorodeoxyglucose ([^18^F]FDG) as a radiotracer to detect sites of inflammation (either due to bacterial infection or primary inflammation) has led to exploring the role of PET in visualizing bacteria directly at sites of infection. However, the results from such efforts are controversial and inconclusive so far. We aimed to assess the limitations of PET as an effective modality in the diagnosis of bacterial infections. Inflammation due to bacterial infections can be visualized by using [^18^F]FDG-PET. However, the non-specificity of [^18^F]FDG makes it undesirable to visualize bacteria as the underlying cause of inflammation. Hence, more specific radiotracers that possibly bind to or accumulate in bacteria-specific receptors or enzymes are being explored. Several radiotracers, including 2-deoxy-2-[^18^F]fluorosorbitol ([^18^F]FDS), 6-[^18^F]-fluoromaltose, [^11^C]para-aminobenzoic acid ([^11^C]PABA), radiolabeled trimethoprim (^11^C-TMP) and its analog fluoropropyl-trimethoprim (^18^F-FPTMP), other radiolabeled sugars, and antimicrobial drugs have been used to image microorganisms. Unfortunately, no progress has been made in translating the results to routine human use; feasibility and other factors have constrained their success in clinical settings. In the current article, we discuss the limitations of direct bacterial visualization with PET tracers, but emphasize the important role of [^18^F]FDG-PET as the only option for detecting evidence of infection.

## 1. State of PET Imaging in Infectious Diseases

**Figure 1:**
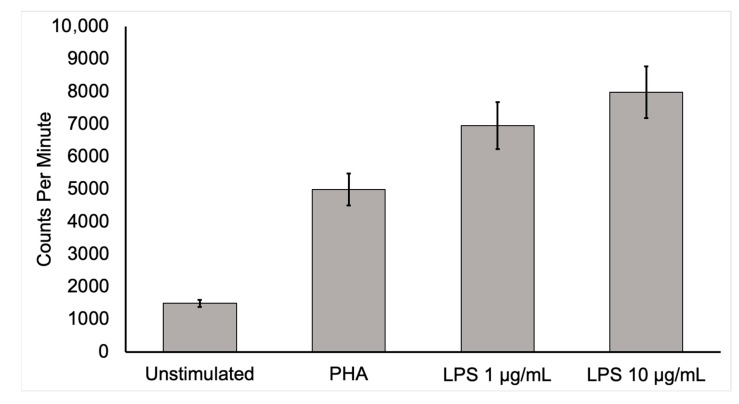
Activated mononuclear cell deoxyglucose uptake. To show that activated inflammatory cells have a higher uptake of [^18^F]FDG, human mononuclear cells from a healthy, adult male were isolated and cultured for 6 h in media containing [^3^H]deoxyglucose ([^3^H]DG) in the absence of stimulants and in the presence of lipopolysaccharide (LPS) or phytohemagglutinin (PHA). After culture, samples were washed three times in phosphate-buffered saline, collected, and placed in a scintillation counter. Mononuclear cells uptake of [^3^H]DG was several times more in the stimulated state than in the unstimulated control condition. This in vitro study supports the conclusion that active inflammatory cells dramatically increase [^18^F]FDG uptake. Moreover, highly increased [^18^F]FDG uptake by activated inflammatory cells at infection sites is likely to allow the detection of infection by this technique.

In 1976, when [^18^F]-fluorodeoxyglucose ([^18^F]FDG) was introduced as a radiotracer for positron emission tomography (PET), it revolutionized medical imaging, especially in the fields of neurology, oncology, and cardiology [1,2,3]. Later, it also gained importance in diagnosing infectious and inflammatory disorders [4,5]. [^18^F]FDG, as an analog of glucose, accumulates in a cell with high rates of glycolysis (such as in cancer cells and inflammatory cells) by entering the cell via glucose transporters and is then phosphorylated by hexokinase to deoxyglucose phosphate, which remains locked in this state [6]. The high uptake of [^18^F]FDG by the metabolically active inflammatory cells has played a major role in the detection of inflammatory reactions in response to microorganisms such as bacteria. Hence, [^18^F]FDG is commonly used for detecting infectious and inflammatory disorders [7] (Figure 1). 

Interestingly, [^18^F]FDG, as a tracer to detect and characterize infections and inflammatory disorders, has been considered to be a major drawback since it leads to false-positive results in patients with cancer [7]. However, over recent years, [^18^F]FDG has been adopted as a powerful modality for detecting sites of inflammation including bacterial infections [7]. Currently, it is well established that inflammatory cells such as neutrophils and macrophages have a high concentration of glucose transporters in their cell membranes, enhancing cellular glucose metabolism [7]. Furthermore, circulating cytokines during inflammation also seem to increase the affinity of these transporters [7]. Hence, [^18^F]FDG remains to be one of the most studied and commonly used radiotracers for diagnosing human infection and inflammation [4]. Due to its versatility, [^18^F]FDG has been appropriately referred to as the ‘‘molecule of the century’’ owing to its enormous impact on the day-to-day practice of medicine [8].

**Figure 2:**
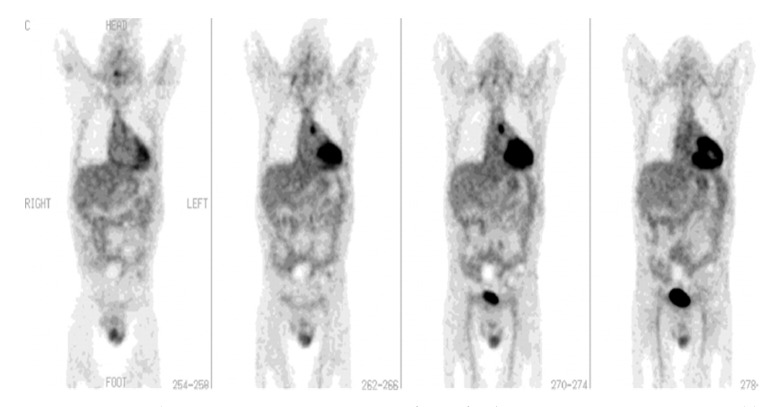
[^18^F]FDG-PET imaging in fever of unknown origin (FUO). A 44-year-old man after heart transplant presented with fever of unknown origin and inconclusive radiologic studies, including CT. Coronal PET images demonstrate a focus of increased [^18^F]FDG activity in the aortopulmonary window that represents the source of infection. The patient completely recovered following drainage of the infected site in the mediastinum (with permission from [10]).

With the introduction of combined PET/computed tomography (CT) in 2001, PET/CT has become one of the most widely used imaging techniques for diagnosing infectious and inflammatory disorders [4]. However, [^18^F]FDG, as the molecular imaging test of choice for many inflammatory and infectious indications (including sarcoidosis, fever of unknown origin, and musculoskeletal infection), was only recently approved by the Centers for Medicare and Medicaid Services (CMS) in the United States [9]. All along, there has been a growing interest in exploring the usefulness of [^18^F]FDG-PET/CT in many infectious and inflammatory disorders beyond its original research trials [10]. The clinical use of PET imaging is being widely studied for chronic osteomyelitis, complicated lower-limb prostheses, complicated diabetic foot, fever of unknown origin (Figure 2), acquired immunodeficiency syndrome (AIDS), vascular graft infection, and fistula, among various other indications [10]. 

### 1.1. State of [^18^F]FDG-PET Imaging in Fever of Unknown Origin

Fever of unknown origin (FUO) was defined in 1961 as a disease condition where body temperature exceeds 38.3 °C on at least three occasions over three weeks, with no diagnosis made despite one week of investigations in the hospital [11]. In the report by Petersdorf and Beeson, the causes of FUO with more than 200 identified diagnoses were classified as infection (36%), malignancy (19%), collagen vascular diseases (19%), and miscellaneous (19%), with no cause found in some cases (7%) [12]. 

Although it was defined and classified more than 50 years ago, FUO still presents a challenge in diagnosis due to the lack of a specific diagnostic algorithm. The wide range of clinical presentations with diversity in probable causes has also added to the challenge in diagnosis. [^18^F]FDG-PET/CT, with its ability to detect both metabolic and structural details of the cause of FUO, can be used as the diagnostic modality of choice for FUO [11]. Furthermore, as metabolic changes occur earlier than morphological changes during inflammation, [^18^F]FDG PET/CT also has the added benefit of identifying areas of inflammation at their early stages as compared with other diagnostic modalities [13]. Moreover, the recently introduced, total-body PET imaging has the additional advantage of increased sensitivity even with a relatively low radiation exposure when compared to a CT scan. [^18^F]FDG-PET has been shown to have a high sensitivity in the workup of FUO [14]. The diagnostic accuracy of [^18^F]FDG-PET/CT reaches 89% when performed in cases of FUO with increased c-reactive protein (CRP) and erythrocyte sedimentation rate (ESR) levels [15]. A retrospective study also found that [^18^F]FDG-PET/CT was used in the confirmation of suspected causes of FUO in 56.6% of cases, with infection accounting for 21%, malignancy accounting for 22%, noninfectious inflammatory diseases accounting for 12%, others accounting for 5%, and the cause unknown in 40% [16,17]. To date, there has been a very wide range of applications of [^18^F]FDG-PET/CT. It has been utilized in the detection of infective endocarditis [16] as well as prosthetic valve endocarditis [16,17]. Furthermore, it has been applied in the diagnosis of sarcoidosis [18] and cranial giant cell arteritis [19]. 

Two studies were conducted to evaluate the effectiveness of [^18^F]FDG-PET/CT in diagnosing FUO. The first study, a meta-analysis, found that using [^18^F]FDG-PET/CT resulted in a high rate of negative predictive values and improved the overall diagnostic rate for FUO [5]. The second study, conducted by Pereira et al., found that [^18^F]FDG-PET/CT was able to confirm the cause of FUO in 56.6% of cases, with causes ranging from infection, malignancy, and non-infectious inflammatory disease to other factors and unknown causes. These studies indicate that [^18^F]FDG-PET/CT is an effective tool for diagnosing FUO and may provide more accurate diagnoses in many cases.

In many recent studies, [^18^F]FDG-PET/CT has proven to be a highly sensitive diagnostic tool for FUO. When we compare it to other conventional diagnostic modalities used currently for the diagnosis of FUO, it has been found to have better sensitivity and specificity, along with its use in the detection and localization of the lesions [5,13,20]. Furthermore, it can be adapted for use in monitoring and evaluating the treatment response. [^18^F]FDG-PET/CT is comparably inexpensive compared to other nuclear imaging studies and has the advantage of providing results on the same day; hence, it can be more cost-effective as it can help to avoid unnecessary invasive tests while decreasing the hospital stay [15,21].

### 1.2. State of [^18^F]FDG-PET Imaging in Cardiovascular Infections

**Figure 3:**
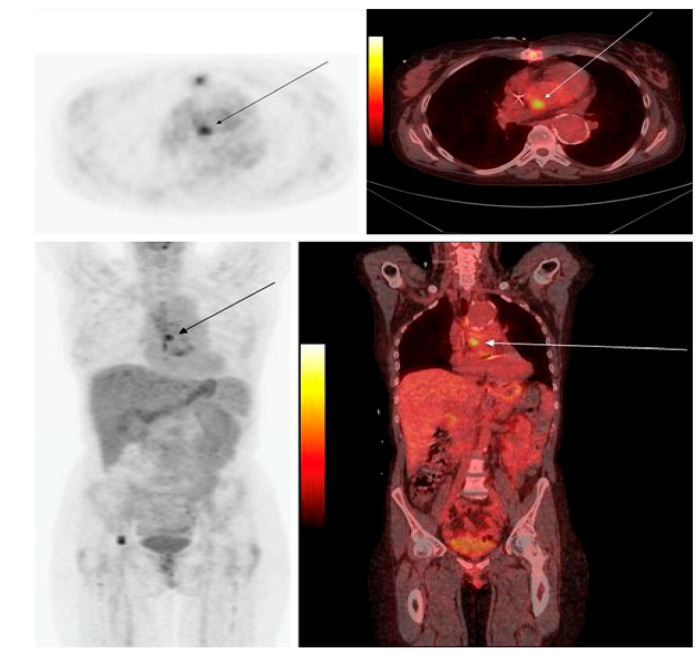
PET/CT in infective endocarditis. The PET/CT image of a 47-year-old female with IE: (**Upper**) transaxial image; (**lower**) coronal image, with PET on the left and the PET/CT fusion image on the right. The images depict a focal region of increased [^18^F]FDG uptake in the heart at the position of the valvula aorta (with permission from reference [34]).

[^18^F]FDG-PET/CT has been found to play a role in the evaluation of endocarditis, myocarditis, and pericarditis. Transthoracic echocardiography along with blood culture has traditionally been a diagnostic modality of choice for detecting cardiovascular infections such as infective endocarditis (IE) [22]; IE poses a diagnostic dilemma due to its very diverse clinical presentation. The current method of diagnosis uses modified Duke criteria (MDC), which are divided into “major criteria” (typical blood culture and positive echocardiography) and “minor criteria” (predisposition, fever, vascular phenomena, immunologic phenomena, suggestive echocardiogram, and suggestive microbiologic findings). However, it creates a problem for patients with equivocal clinical symptoms in the absence of conventional echocardiographic features (particularly when prosthetic heart valves are present), making the diagnosis difficult [23]. Imaging modalities such as transesophageal echocardiogram (TEE), CT, and magnetic resonance imaging (MRI) have been studied. However, a number of technical factors, which include the presence of prosthetic heart valves and the aortic graft, prevent these imaging modalities from being accurate and reliable. PET/CT has demonstrated an advantage over echocardiography (Figure 3), especially in prosthetic valve endocarditis, but its role in native valve endocarditis is still unclear [24,25,26]. In such patients, when [^18^F]FDG-PET/CT is combined with MDC, the sensitivity of IE diagnosis appears to increase [27,28]. Additionally, there has also been an improvement in the diagnosis of symptomatic or asymptomatic septic embolism [28,29,30,31]. The detection of a septic embolism has also helped to change the therapeutic decision as its presence necessitates a longer duration of antibiotic treatment or timely surgical consultation. Furthermore, compared to PET/CT, PET/CT-angiography is able to detect considerably more abscesses and collections, as well as numerous lesions that are important for clinical and surgical decision-making [32]. However, leukocyte scintigraphy appears to be advantageous over [^18^F]FDG-PET/CT in the first 2 months post open cardiac surgery due to the possibility of a high and comparable level of radiotracer uptake in the inflammatory tissues [33].

The most common cause of myocarditis is infection, especially viral infections [22]. Creatine kinase MB-muscle and brain (CK-MB) and troponin-I have high specificity but lack sensitivity in diagnosing myocarditis [35,36]. Similarly, echocardiography also has less sensitivity and can show either normal heart function or global/regional left ventricular hypokinesis [37]. [^18^F]FDG-PET/CT can be a useful diagnostic tool as it can demonstrate increased metabolic activity in the myocardium [38]. Radiation exposure also decreases with PET/MR as compared to PET/CT. [^18^F]FDG-PET findings could provide complementary and additive benefits to cardiac MR by increasing sensitivity for mild or borderline myocarditis and increasing specificity for chronic myocarditis [38,39].

Viral pericarditis is the most common cause of acute pericarditis. The presence of associated pericardial effusion and concomitant myocarditis can be detected using echocardiography and cardiac MRI [40]. [^18^F]FDG-PET/CT can also detect the inflammation correlating with cardiac MRI in those cases [41]. However, with quick assessment and decision-making, CT and echocardiography are more beneficial in the diagnosis of viral, bacterial, and fungal pericarditis than PET/CT [42]. Interestingly, [^18^F]FDG-PET/CT can be superior to CT in detecting tuberculous pericarditis [43]. As shown in a study of nine patients, dual-phase [^18^F]FDG PET/CT identified 18 sites of associated lymph node involvement, among which 9 sites were not identified on CT [43]. Furthermore, [^18^F]FDG-PET/CT can also be useful in the diagnosis of metastatic infection in purulent pericarditis with septicemia [42].

### 1.3. Role of [^18^F]FDG-PET/CT in Musculoskeletal Infections

Imaging methods are part of the diagnostic workup for musculoskeletal infections, which are often challenging diagnoses. Although gallium-67, labeled leukocytes, and bone imaging with radionuclides are the most often used techniques in this context, [^18^F]FDG-PET/CT may play an essential role in the clinical diagnosis of acute, subacute, and chronic bone marrow and soft tissue infections. Compared to traditional radionuclide procedures, [^18^F]FDG-PET/CT offers the advantage of locating abnormalities more precisely, and monitoring response to treatment [44].

**Figure 4:**
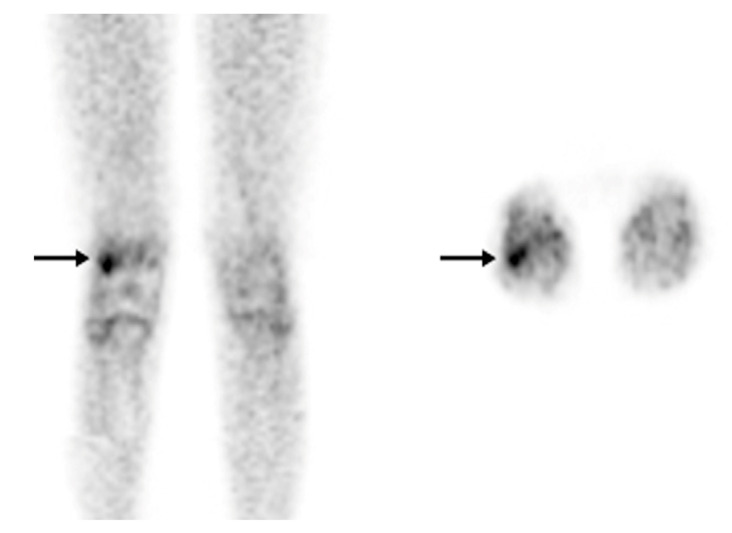
[^18^F]FDG-PET in osteomyelitis. Osteomyelitis of the right femur. An [^18^F]FDG-PET scan was recommended for a 12-year-old patient with bacteremia and right knee pain instead of labeled leukocyte imaging due to leukopenia. On the coronal (**left**) and axial (**right**) images, there is focal hypermetabolism (SUV max: 2.5) in the lateral condyle of the right femur (arrow) (with permission from [44]).

The role of [^18^F]FDG-PET/CT has been found to be promising in a number of musculoskeletal infectious disorders. [^18^F]FDG-PET/CT is important for diagnosing persistent musculoskeletal infections [45] including the detection of chronic osteomyelitis [46] (Figure 4). Some other uses of [^18^F]FDG-PET/CT may include evaluation of the diabetic foot [47], implant-related infections in the leg [48], and septic arthritis [44,49,50,51,52]. 

Numerous molecular imaging techniques have been used to diagnose and evaluate treatment responses in patients with osteomyelitis. Commonly used radiopharmaceuticals such as combined bone marrow/leukocyte scintigraphy, gallium scintigraphy, combined [^99m^Tc]-methyl diphosphonate ([^99m^Tc]MDP) bone/gallium scintigraphy, and combined [^99m^Tc]MDP bone/leukocyte scintigraphy have significant limitations in this context, which can be overcome by using [^18^F]FDG PET/CT [53]. [^18^F]FDG-PET has shown higher sensitivity (96%) and specificity (91%) in chronic osteomyelitis compared to a bone scan, leukocyte scan, and a combined bone/leukocyte scan and MRI [10]. Moreover, when it comes to differentiating chronic osteomyelitis (duration > 6 months) from aseptic post-operative/traumatic bone healing, [^18^F]FDG-PET/CT plays an important role. [^18^F]FDG uptake persists in chronic osteomyelitis, since activated macrophages continue to accumulate [^18^F]FDG in chronic infection [54,55]. 

One of the important domains where [^18^F]FDG-PET/CT is definitely helpful is in the diagnosis of spinal osteomyelitis. [^18^F]FDG-PET has higher diagnostic accuracy for the detection of vertebral chronic osteomyelitis compared to a leukocyte scan [10]. In addition, [^18^F]FDG-PET has the advantage of being less susceptible to attenuation or metal artifacts due to implants compared to structural imaging modalities [56]. However, care must be taken while differentiating chronic osteomyelitis from false positive results on [^18^F]FDG-PET/CT due to fractures, inflammatory arthritis, or normal bone healing after surgery. Due to its high negative predictive value, [^18^F]FDG-PET/CT has also been found to be a useful addition to MRI for differentiating degenerative and infectious end plate abnormalities [1]. Notably, degenerative changes exhibit only mildly elevated [^18^F]FDG uptake [57].

The role of [^18^F]FDG-PET/CT in the evaluation of diabetic foot infection remains unclear, with some researchers finding great accuracy and others reporting the exact opposite [44]. It is crucial to distinguish between osteomyelitis in the diabetic foot and neuropathic osteoarthropathy, since their respective treatments differ. Neuropathic osteoarthropathy demonstrates a lower [^18^F]FDG metabolism than osteomyelitis [47,58]. In a study of 39 patients with a clinically suspected diabetic foot infection, [^18^F]FDG-PET/CT demonstrated good sensitivity (100%), specificity (92%), PPV (87%), and NPV (95%). However, the diagnostic accuracy of leukocyte scans was shown to be superior to that of [^18^F]FDG-PET/CT in another study [59]. Presumably, variability in serum glucose level prior to the [^18^F]FDG-PET/CT exam (which is a regular occurrence in diabetic patients) may account for the contradictory results [53].

The role of [^18^F]FDG-PET/CT in prosthetic joint infection is somewhat established but may require further validation [53]. In addition, the role of [^18^F]FDG PET/CT in the clinical differentiation of prosthetic joint infection from displacement/aseptic loosening is also not clear. Peri-prosthetic [^18^F]FDG activity in the prosthesis-bone interface is very specific for infection [60,61] and has high sensitivity and specificity [62,63,64,65]. In contrast, non-specific uptake is seen around the femoral neck as an inflammatory reaction [57]. 

Few studies exist regarding the usefulness of [^18^F]FDG-PET in septic arthritis. [^18^F]FDG accumulates in inflammatory arthritis, and its diagnostic usefulness in septic arthritis is likely limited [44,49,50,51,52].

## 2. Limitations of PET for Direct Visualization of Bacteria

Over the past three decades, efforts have been made to develop single-photon emission computerized tomography (SPECT) and PET tracers that will target bacteria, and therefore, differentiate between pure inflammation and infectious disorders [10,66,67,68,69,70,71,72,73,74]. In contrast to imaging techniques such as CT and MRI, the spatial resolution of PET imaging is still suboptimal for portraying details related to targeted structures, despite significant advances that have been made in recent years. While the spatial resolution of CT and MRI is in the range of 1–2 mm, that of PET is substantially worse (in the range of 5–10 mm) in human imaging studies [8,74,75]. This is mainly due to the basic limitations of this technology but also relates to physiologic factors such as motion and duration of image acquisition. While PET images of organs such as the brain reveal optimal details for assessing certain disorders, the modality faces substantial challenges in the trunk due to various physical and physiological factors [75]. These limitations are unavoidable in spite of the introduction of specific compounds designed to detect and characterize certain diseases and disorders. 

Therefore, extrapolating what has been achieved in the in vitro setting or by adopting autoradiographic imaging approaches to in vivo human studies is somewhat naïve and unrealistic. In order to detect microscopic structures such as bacteria at the sites of infection, realistically, it would be necessary to accumulate a large volume in the range of 8–10 cubic mm for detection by PET imaging. Furthermore, the concentration of imaging agents by these microorganisms should be significantly higher than that of the background to reach appropriate contrast compared to surrounding background activity [7,66,73,74,75,76].

Since bacteria are rapidly phagocytized by white blood cells that are attracted to sites of infection, they are not exposed to radiotracers that reach infected sites. In other words, it is unlikely that a large volume of bacteria will accumulate freely (without phagocytosis) to a certain size (several mm) before being attacked by the white blood cells that infiltrate these sites. This phenomenon will be an ongoing process in most bacterial infections. Therefore, it is unlikely that a large volume of bacteria will be exposed to the radiotracers that have been successfully synthesized based on in vitro testing [66,74,77].

This limitation of PET is not only applicable to detecting bacteria in vivo successfully, but it is also relevant to its role in several other domains [75,78]. These claims and such applications of PET are unjustified and can lead to inappropriate use of this powerful technology by the medical community.

In spite of these limitations, multiple compounds have been tested for direct visualization of bacteria with conflicting results.

## 3. Specific Radiotracers Studied for Direct Visualization of Bacteria

**Figure 5:**
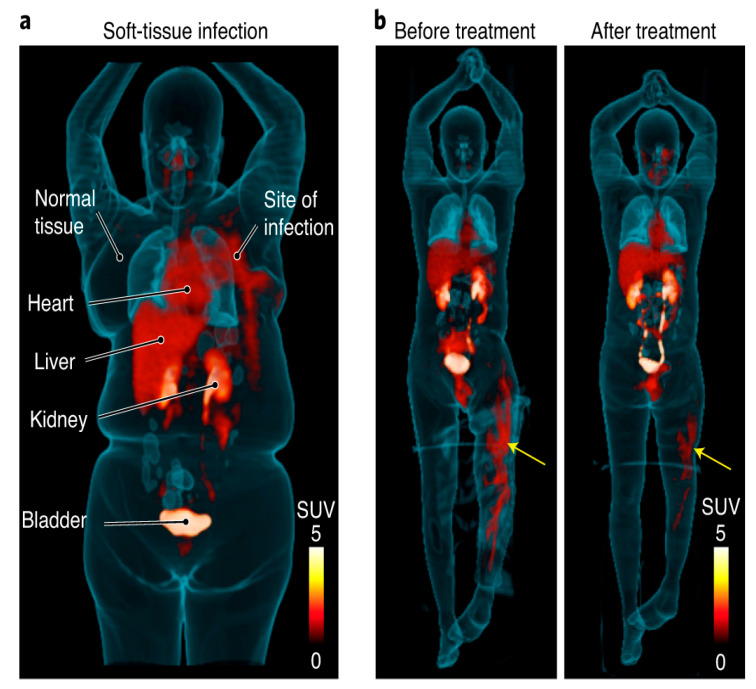
[^18^F]FDS PET/CT imaging in patients with confirmed Enterobacterales infections. (**a**) Three-dimensional maximum intensity projection (MIP) from a patient with microbiologically confirmed *Enterobacter aerogenes* cellulitis of the left breast. Signal is also noted in the heart (blood pool), liver, kidneys, and the urinary bladder. (**b**) Three-dimensional MIP from a patient with MDR, extended spectrum beta-lactamase (ESBL)-producing *E. coli* osteomyelitis before and after inadequate treatment. Yellow arrows indicate site of infection (with permission from [88]).

Several approaches toward the direct detection of bacteria have been put forward to develop specific radiotracers, including radiolabeled antibiotics, antibodies, antimicrobial or chemotactic peptides, and even bacteriophages [79,80]. For example, [^68^Ga]Ga-desferrioxamine-B ([^68^Ga]Ga-DFO-B) and [^68^Ga]Ga-pyoverdine PAO1 ([^68^Ga]Ga-PVD-PAO1) are radiolabeled siderophores which were developed in order to target bacterial transporters [81,82]. Peptides and amino acids which demonstrate accumulation in bacteria, such as D-[methyl-^11^C]methionine ([^11^C]D-Met) and [^68^Ga]Ga-NOTA/DOTA-UBI-29-41, have also been proposed as potential tracers used in PET imaging of infection [83,84,85]. In addition, [^18^F]FDS (Figure 5) and 6-[^18^F]-fluoromaltose have been investigated as alternative sugar-based radiotracers more specific to bacterial activity compared to [^18^F]FDG [86,87,88], which is taken up by bacterial and human cells alike. However, the results obtained so far have yet to demonstrate clinical utility. It is very likely that some of the positive results that have been reported with these bacterial agents are the result of hyperemia at the sites of bacterial infection; nonetheless, these results have been misunderstood as proof of the binding of these agents to bacteria.

Bacteria synthesize folate by incorporating para-aminobenzoic acid (PABA) and pteridine with the help of bacterial dihydropteroate synthase, an enzyme not present in human cells. As such, this pathway has been considered to be a possible target for pathogen-specific imaging of bacteria [89]. PET imaging with [^11^C]PABA has been proposed to image MRSA, targeting the folate synthesis pathway. Inhibitors such as radiolabeled trimethoprim ([^11^C]TMP) and its analog fluoropropyl-trimethoprim ([^18^F]FPTMP) are also being studied as bacteria-specific imaging agents due to their n1anomolar affinity for key enzymes in the folate synthesis pathway [89].

The nucleoside analog fialuridine (FIAU) serves as a substrate for thymidine kinase in bacteria (TK) [90]. A study was conducted to see if [^124^I]FIAU PET/CT could be effectively used for a PJI diagnosis with sufficient precision. However, the clinical usefulness of [^124^I]FIAU for the detection of PJIs was found to be limited due to poor image quality and low specificity [91].

### Antibiotic Tracers for the Evaluation of Bacterial Infections

Antibiotic tracers represent another method proposed to evaluate bacterial infection [7,79,92,93,94]. Studies have investigated quinolones as potential PET tracers in this domain. However, in vitro experiments using these potential tracers have revealed nonspecific absorption in the presence of excess unlabeled chemicals and their binding to heat-killed bacteria, severely limiting its optimal utility for clinical applications [92]. Likewise, the extremely low concentration at which antibiotics or antimicrobial peptides kill or disable bacteria also limits their use as a radiotracer due to a lack of signal amplification [89]. It must be noted that the use of antimicrobials to detect bacteria has several disadvantages. Antibiotic resistance is of rising concern as the results will be futile if the target bacteria is resistant to the antibiotic tracer used in PET [95,96]. 

Although metabolic agents and radiolabeled sugars have shown potential in small animal trials and with a few bacterial species, they do not seem to have the same broad-spectrum bacterial affinity as TMP and have a significant background uptake of normal tissues [86,97,98,99]. 

## 4. Conclusions

Although bacteria-specific PET radiotracers may appear to have some potential for diagnosing bacterial infections directly, this endeavor has had little success over the past decades. This is primarily due to limitations of PET as a high-resolution technique and the biological sequences that bacterial infections follow during the course of the disease. Therefore, [^18^F]FDG-PET will remain to be the imaging modality of choice in assessing various infections. This is particularly true in assessing patients with musculoskeletal infections. The arrival of total body PET will allow for simultaneous evaluation of the entire body for occult infection, vastly increasing the role of molecular imaging in difficult clinical cases. Given the prominent role of MRI in assessing infections such as osteomyelitis, PET/MRI may have a future role to play as well by combining the strengths of these two modalities in assessing different aspects of infectious processes. Therefore, PET imaging of infections with [^18^F]FDG has the potential to expand and develop alongside developments in hybrid imaging technology. 

## Data Availability

Not applicable.
